# A diverse set of Enterococcus-infecting phage provides insight into phage host-range determinants

**DOI:** 10.1016/j.virusres.2024.199426

**Published:** 2024-07-04

**Authors:** Alhassan M. Alrafaie, Karolina Pyrzanowska, Elspeth M. Smith, David G. Partridge, John Rafferty, Stephane Mesnage, Joanna Shepherd, Graham P. Stafford

**Affiliations:** a Department of Medical Laboratory, College of Applied Medical Sciences in Al-Kharj, Prince Sattam Bin Abdulaziz University, Al-Kharj 11942, Saudi Arabia; b School of Clinical Dentistry, University of Sheffield, Sheffield, United Kingdom, S10 2TA, UK; c School of Biosciences, University of Sheffield, Sheffield, S10 2TN, UK; d Sheffield Teaching Hospitals NHS Foundation Trust, Sheffield S10 2JF, UK

**Keywords:** Bacteriophages, Enterococcus, Antibiotic resistance, Enterococcal polysaccharide antigen (EPA)

## Abstract

Enterococci are robust Gram-positive bacteria that pose a significant threat in healthcare settings due to antibiotic resistance, with vancomycin-resistant enterococci (VRE) most prominent. To tackle this issue, bacteriophages (bacterial viruses) can be exploited as they specifically and efficiently target bacteria. Here, we successfully isolated and characterised a set of novel phages: SHEF10, SHEF11, SHEF13, SHEF14, and SHEF16 which target *E. faecalis* (SHEF10,11,13), or *E. faecium* (SHEF13, SHEF14 & SHEF16) strains including a range of clinical and VRE isolates. Genomic analysis shows that all phages are strictly lytic and diverse in terms of genome size and content, quickly and effectively lysing strains at different multiplicity of infections. Detailed analysis of the broad host-range SHEF13 phage revealed the crucial role of the enterococcal polysaccharide antigen (EPA) variable region in its infection of *E. faecalis* V583. In parallel, the discovery of a carbohydrate-targeting domain (CBM22) found conserved within the three phage genomes indicates a role in cell surface interactions that may be important in phage-bacterial interactons. These findings advance our comprehension of phage-host interactions and pave the way for targeted therapeutic strategies against antibiotic-resistant enterococcal infections.

## Highlights

• Five novel enterococcal phages were isolated and characterized with varied host range.
• SHEF14 has a small genome (19.3 Kb) and effectively infects a VRE strain.
• Enterococcal polysaccharide antigen (EPA) has a crucial role for SHEF phage infection.
• A carbohydrate-targeting domain (CBM22) was found conserved within three phage genomes.

## 1 Introduction

Enterococci are Gram-positive bacteria commonly found in the gastrointestinal tracts of humans and many animals. They are frequently isolated in hospital settings due to their resilience and adaptability (Gilmore et al., 2013). Two enterococcal species (*Enterococcus faecalis* and *E. faecium*) are commonly isolated in both hospital and community-acquired infections. These cause urinary tract infection (UTI), endocarditis, bacteraemia, intravenous line and wound infections as well as in patients with diabetic foot ulcers (DFU) (Fiore et al., 2019; Perzon et al., 2023). Due to the growing prevalence of antibiotic resistant strains such as vancomycin resistant enterococci VRE (also referred to as glycopeptide resistant enterococci (GRE)), these infections pose a significant challenge and are often difficult to treat. The World Health Organization (WHO) marked VRE as a high-priority pathogen on pathogens list for research and development of new antibiotics (“WHO Publishes List of Bacteria for Which New Antibiotics Are Urgently Needed,” 2017).

One major contributor to how Enterococcus strains direct host-pathogen interactions involves its surface Enterococcal polysaccharide antigen (Epa) which can also play a crucial role as a phage receptor, as shown by several groups in phage-host studies in Enterococcus spp. (Chatterjee et al., 2019; Al-Zubidi et al., 2019; Canfield et al., 2021). The EPA is anchored to peptidoglycan and comprises conserved and variable regions. It's conserved region is encoded by a conserved genetic locus and is chiefly a polymer of alpha-linked rhamnose sugars linked to glucose or glcNAc residues in a repeating unit (Guerardel et al., 2020). The variable region meanwhile exhibits significant strain diversity, at least as indicated by strain genetics, and in the cases where it has been elucidated contains repeats of teichoic acid like units comprising ribitol, phosphate, GalNAc and glucose (Guerardel et al., 2020).

Bacteriophage (‘phage’) are viruses that specifically target bacteria and are arguably the most abundant biological entities on the planet (Clokie et al., 2011). They were first discovered over 100 years ago by Frederick Twort (Twort, 1915) and Félix d'Herelle (Lourenço et al., 2018) and then quickly applied to kill pathogenic bacteria (Duckworth, 1976). However, the discovery of antibiotics reduced research into the use of bacteriophage, except for limited geographical areas such as Poland and Georgia (Clokie et al., 2011). Given the escalating problem of antibiotic resistance, interest in phage therapy has seen a resurgence to tackle various antimicrobial resistant (AMR) bacterial infections (Khalifa et al., 2015; Paul et al., 2021; Vehreschild et al., 2019). Bacteriophage therapy has attracted interest as an effective alternative or potentiator for antibiotics because it provides specificity in targeting bacterial strains and lowers the risk of disrupting the balance of the natural microbiota (Ganeshan and Hosseinidoust, 2019).

Utilising the therapeutic potential of phages requires the identification and characterisation of an ever-broader range of phage that can attack and lyse pathogenic AMR bacterial strains. Building on our previous work (Al-Zubidi et al., 2019), this manuscript focuses on isolating and characterising bacteriophage from Sheffield wastewater treatment plants that specifically target VRE and clinical *E. faecalis* and *E. faecium* strains from patients with diabetic foot infections. We characterised our latest SHEF phages in terms of morphology, host range, genomic analysis and killing efficacy and this data shows the potential application of SHEF phages to tackle enterococcal strains including VRE.

## 2 Methods

### 2.1 Bacterial strains

All strains of *E. faecalis* or *E. faecium* strains used for phage isolation, host range analysis and mutant studies are listed in Table 1. Cells were grown at 37 °C in Brain heart infusion (BHI). Saline magnesium (SM) buffer (50 mM Tris–HCl, pH 7.5, 100 mM NaCl,8.0 mM MgSO4, 0.01 % (w/v) gelatin) or BHI broth were used for phage storage at 4 °C. Strains *E. faecalis* V583 Δ*epa_var* and *E. faecalis* OG1RF Δ*epa_var* as well as the pIL plasmids containing the *epa_var* region (EF2164-EF2177, Genbank accession: AE016830.1) region were a kind gift from Prof P Serror. The OG1RF Δvar strain was built using the same strategy previously described for the V583 Δvar mutant (Furlan et al., 2019). Since the homology regions upstream and downstream of the variable region are almost identical, allelic exchange could be carried out in OG1RF using plasmid pVE1483 (Furlan et al., 2019). Briefly, OG1RF was electroporated with pVE1483 and transformants were selected at 28 °C on BHI-agar plates containing 30 µg/ml Erythromycin. Individual colonies were re-streaked on BHI-agar in the presence of Erythromycin at 42 °C to screen for recombinant clones that had undergone a single crossing over at the epa locus (large colonies). Two independent candidates were grown in 50 ml of BHI broth without antibiotics and sub-cultured five times overnight (50 µl inoculum in 50 ml). Cells were plated on BHI-agar and screened for the loss of Erythromycin resistance. Positive candidates were analysed by PCR to confirm the deletion of the entire epa_var locus. For heterologous complementation experiments, a pIL252 derivative encoding the entire epa_var locus was built by Gibson assembly. pIL252 was PCR amplified using oligonucleotides OEF904 and OEF1015; the epa locus was amplified using 3 overlapping independent PCR products using oligonucleotides OEF1010 and OEF904 (fragment 1), OEF1011 and OEF1012 (fragment 2), OEF1013 and OEF1014 (fragment 3). The four PCR products were purified and assembled using the NEB HiFi kit according to manufacturer's instructions. The corresponding assembly product was directly used to transform the OG1RF Δepa_var strain and two positive clones growing on BHI-agar supplemented with 3 µg/ml Erythromycin were used for further analysis. pIL_O plasmid was extracted and sequenced to verify the sequence.

**Table 1:** A summary list of enterococcal strains used in this study (a full detailed list of strains is included in Supplementary Table 1).

Strain*	**Species**	**Source**	**Reference**
DP1, DP2, DP3, DP4, DP5	*E. faecalis*	Clinical isolate (DFU)	Dr. David Partridge
DP6, DP7, DP8, DP9	*E. faecium*	Clinical isolate (DFU)	Dr. David Partridge
E1636 (V1)	*E. faecium*	Clinical isolate (Blood)	(De Been et al., 2013)
E1679 (V1)	*E. faecium*	Clinical isolate (vascular catheter)	(De Been et al., 2013)
E1071 (V2)	*E. faecium*	Clinical isolate (faeces)	(De Been et al., 2013)
E4452 (V2)	*E. faecium*	Clinical isolate (faeces)	(De Been et al., 2013)
EF54	*E. faecalis*	Nonoral human isolate	(Toledo-arana et al., 2001)
JH2–2	*E. faecalis*	Nonoral human isolate	(Jacob & Hobbs, 1974)
V583	*E. faecalis*	Clinical isolate (Blood)	(Sahm et al., 1989)
V583 *ΔepaV*	*E. faecalis*	Deletion mutant of epa variable region (*epaV*)	P. Serror
V583 *ΔepaV + pILepa-V*	*E. faecalis*	Complementation strain containing the V583 *epaV* region cloned into pIL plasmid	P. Serror
OG1RF	*E. faecalis*	Oral human isolate	(Bourgogne et al., 2008)
OG1RF *ΔepaV + pIL-epaV*	*E. faecalis*	Complementation strain containing the V583 *epaV* region cloned into pIL plasmid	Prof P. Serror

⁎ V1–4 indicates EPA_var clade types according to (De Been et al., 2013).

### 2.2 Phage isolation

Wastewater samples collected from Sheffield wastewater treatment plants were centrifuged (10,000 xg, 10 min) and then passed through 0.45 mm syringe filters. The filtered samples were then concentrated if necessary using Amicon® Ultra-15 Centrifugal Filter Units (100,000 kDa MWCO). Our isolation methodology involved both single and multiple strain enrichment steps. For the single strain approach, 30 μl of concentrated wastewater samples were mixed with 10 ml of exponentially growing indicator bacteria and incubated overnight at 37 °C with shaking. The enriched sample was then centrifuged (4000 xg, 10 min, 4 °C) and the supernatant re-filtered (0.45 μm) and stored at 4 °C. For multiple strains, concentrated wastewater samples were added to a bacterial culture consisting of 4 strains in mid-log phase according to Hyman (2019) - 1 ml of each culture added to a 26 ml BHI broth containing 1 ml of concentrated wastewater. The mixture was incubated overnight at 37 °C with shaking before being centrifuged (4000 xg, 10 min) and the supernatant filtered again before storage at 4 °C. To assess phage isolation, plaque assays were performed by mixing 200 μl of bacterial culture, 20 μl of filtered enriched supernatants and 4 ml of molten BHI top agar (0.4% w/v) and poured onto BHI agar and incubated at 37 °C overnight. For phage purification, 3 consecutive passages of well-isolated plaques were performed.

### 2.3 Transmission electron microscopy (TEM)

4 μl of phage suspension was placed on carbon-coated copper grids for 5 min and withdrawn using filter papers. The grids were then negatively stained with 2 % (w/v) uranyl acetate (4 μl) pH 4 for 1 min before the stain removed. Particles were imaged digitally using a FEI Tecnai Transmission Electron Microscope at an accelerating voltage of 80 KV, at the electron microscope facility (University of Sheffield, School of Biosciences).

### 2.4 Host range

Strains were tested for phage killing using a double-layer agar spot tests where 5 μl of phage (10^7^ plaque forming units/ml ((PFU/ml)) were deposited on bacterial lawns and incubated at 37 °C. Next day, all spots showing lysis were further confirmed by spotting serially diluted phage 5μl (10^7^,10^6^, 10^5^,10^4^, 10^3^ PFU/ml) on fresh lawns. On the following day, observation of individual plaques indicated phage infection and ruled out ‘lysis from without’. The phage titre was determined by counting the plaques and calculating the efficiency of plating (EOP) in percentage as follows: PFU/ml of phage on the test strain divided by the PFU/ml of phage on the isolating host.

### 2.5 Phage genome extraction, sequencing and analysis

SHEF phage DNA was extracted using the phenol–chloroform–isoamyl alcohol method as described previously (Al-Zubidi et al., 2019). Briefly, 1 μl of DNase I (1 U/μl) and 1μl of RNase A (100 mg/ml) were first added to 1 ml of concentrated phage lysate and incubated at 37 °C for 30 min. Then, 100 μg/ml of proteinase K was added to the mixture and incubated at 50 °C for 45 min. After that, DNA was separated from the denatured proteins by adding phenol: chloroform: isoamyl alcohol (25:24:1). After centrifugation at 14,000 × *g* for 5 min, the aqueous phase was aspirated into a new tube and this step was repeated once. Then, two volumes of ice-cold 100 % ethanol and 1/10 vol of 3 M sodium acetate were added and left overnight at −20 °C to precipitate DNA. Next day, the solution was centrifuged at 16,000 xg for 20 min to pellet DNA and the supernatant was discarded. 70 % ethanol was added, and the tube was centrifuged at 14,000 × *g* for 5 min. The supernatant was discarded, and the tube was left open on the bench for 15 min to air-dry. The pellet was then suspended in 50 μl of autoclaved milli-Q-water and stored at −20 °C. Quality control for genome integrity was assessed using agarose gel electrophoresis. Bacteriophage genomes (50 ng/μl) were sequenced using the Illumina NovaSeq 6000 platform and 250 bp paired-end technology (MicrobesNG, Birmingham, UK). Assembly was performed using SPAdes version 3.7 and annotated was using Prokka 1.13 (Seemann, 2014). SHEF phage genome sequences were deposited at the National Center for Biotechnology Information. Accession numbers are as follows: SHEF10 (OL799256), SHEF11 (OL799257), SHEF13 (OL799258), SHEF14 (OL799259) and SHEF16 (OL799260).

### 2.6 Bioinformatic analysis

Genbank files of the EPA_variable region loci were created using Snapgene software version 7.1 (www.snapgene.com) after reannotation of genomes using the well characterised OG1RF genome as a reference for Prokka (via Galaxy Europe server). Genome regions were compared using clinker: https://cagecat.bioinformatics.nl/tools/clinker (Gilchrist & Chooi, 2021). Genome to genome distance of the isolated phages and related bacteriophage genomes was determined using the Genome-BLAST Distance Phylogeny (GBDP) method under settings recommended for prokaryotic viruses using the VICTOR web service (https://victor.dsmz.de) (Meier-Kolthoff and Göker, 2013, 2017). The resulting intergenomic distances were used to infer a balanced minimum evolution tree with branch support via FASTME including SPR postprocessing (Lefort et al., 2015). Branch support was inferred from 100 pseudo-bootstrap replicates each and rooted at the midpoint (Farris, 1972) . Taxon boundaries at the species, genus and family level were estimated with the OPTSIL program (Göker et al., 2009), using the recommended clustering thresholds (Meier-Kolthoff and Göker, 2017) and an F value (fraction of links required for cluster fusion) of 0.5 (Meier-Kolthoff et al., 2014). Additionally, a phylogenetic tree was generated using MEGA11 based on homology of Major capsid proteins (MCPs) found in the isolated SHEF phages and related phages. The protein sequences were aligned using ClustalW and the phylogenetic tree was created using the neighbour-joining method (Saitou and Nei, 1987).

### 2.7 Killing assays

The impact of phage on planktonic bacteria growth was tested using 200 μl overnight culture inoculum of O.D._600_= 0.05 (2 × 10^6^ CFU) in a 96-well plate incubated overnight at 37 °C with shaking in a Tecan Sunrise Microplate Reader. For assays including phage, 20 μl were added to achieve multiplicity of infections (MOI) of 10, 1 or 0.1. The absorbance readings were then analysed using GraphPad Prism.

## 3 Results

### 3.1 Isolation and characterisation of SHEF phages

Our previous work and that of others (Al-Zubidi et al., 2019; Ho et al., 2018) has indicated that the surface EPA of Enterococcus is an important docking molecule for phage at the cell surface. Enterococcal polysaccharide antigen (EPA) is a rhamnopolysaccharide primarily composed of glucose, rhamnose, N-acetylglucosamine (GlcNAc), N-acetylgalactosamine (GalNAc), and galactose (Hancock and Gilmore, 2002). This molecule has a conserved core glycan region that is decorated with various side chains and modifications that are encoded by a core and variable (var) gene locus in all strains of *E. faecalis* and *faecium* studied to date (Guerardel et al., 2020; Ramos et al., 2021). The epa Var genes are responsible for synthesis and attachment of teichoic acids bound covalently to the rhamnose backbone (Guerardel et al., 2020) as well as synthesis and attachment of other decorating glycans such as polyGlcNAc (Ramos et al., 2019). Of note, in the context of *E. faecium,* analysis of human pathogenic and commensal *E. faecium* strain EPA_var loci has revealed four variants designated based on the composition of their gene loci for EPA_var (De Been et al., 2013). Therefore, to isolate phages targeting a range of EPA-types and with clinical potential we included strains across the four EPA-locus types as well as clinical AMR strains from chronic infected diabetic foot wounds in Sheffield (Table 1 and S2). We obtained environmental samples from Sheffield wastewater treatment plants and processed and enriched them as described.

As part of our strategy, we used single or multiple host enrichments. The single-host approach resulted in isolation of SHEF10 (using *E. faecalis* OS16 strain) and SHEF11 (using *E. faecalis* EF2 strain). For a multiple-host approach, various strains of the four *E. faecium* EPA-locus types were mixed in different combinations, allowing isolation of phages SHEF13, 14, and 16 which all target the VRE *E. faecium* E1071 (type 2 EPA_var locus, EPA_V2) (De Been et al., 2013).

**Fig. 1:**
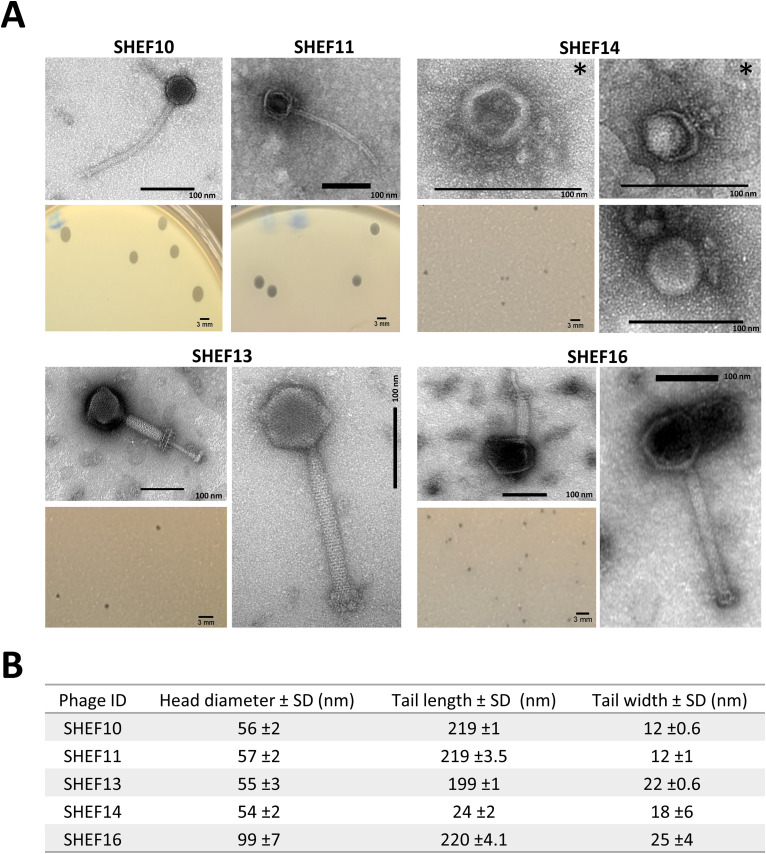
A) Phage and plaque morphologies of SHEF phages. Scale bar is 100 nm for micrographs and 3 mm for plaque sizes. For the contractile SHEF13 and SHEF16 uncontracted and contracted forms are shown while two forms of SHEF14 were also observed, one with a protruding spike (*). B) Dimensions of phage: head diameter, Tail length and width of uncontracted tails from 3 particles are shown (with SD).

All phages produced clear plaques, with SHEF10 and 11 making large plaques (3–4 mm) while SHEF13,14 and 16 plaques were much smaller (0.5–1 mm) (Fig. 1). Following purification, phage virion morphology was assessed by transmission electron microscopy to reveal that SHEF10 and 11 have icosahedral heads and non-contractile tails (219±3.5 nm x 12±1 nm). In contrast, contractile tails of 199±1–220±4.1 nm x 22–25 nm (myoviruses) were seen for SHEF13 and 16 with relaxed and contracted forms observed. Lastly, SHEF14 is a podovirus with an icosahedral head, attached to a short tail (24±2 nm) encased in a wide baseplate that seems to occur in two forms (Fig. 1).

A total of 36 enterococcal strains including clinical isolates from patients with DFU were then tested in host range assays. Spot and plaque assays were performed and the efficiency of plating was determined (Table 2). SHEF10 and 11 only infect *E. faecalis* strains but have distinct host ranges. SHEF10 showed activity against 7 strains including OG1RF and the clinical isolate DP5 (ST 16). SHEF13, on the other hand, demonstrated a broader host range by infecting both vancomycin resistant *E. faecalis* (including V583) and *E. faecium* strains E1071 (ST 32, EPA_V2) as well as the clinical VRE isolate DP9 (ST 787). In contrast, SHEF14 has a narrow host-range infecting only it's isolation strain of *E. faecium* E1071. For SHEF16, infection of E1071 was observed, but also the EPA-V1 strains E1636 and 1679, i.e. a different host-range to SHEF13. It's worth noting that no phages were isolated from the other EPA variants during this process.

**Table 2:** Host range of SHEF phages. Vancomycin Resistant Enterococci are highlighted in red text. Boxes are shaded as shown in the key.

EOP > 0.01  EOP < 0.01)  No lysis.

### 3.2 Genomic and phylogenetic analysis

**Fig. 2:**
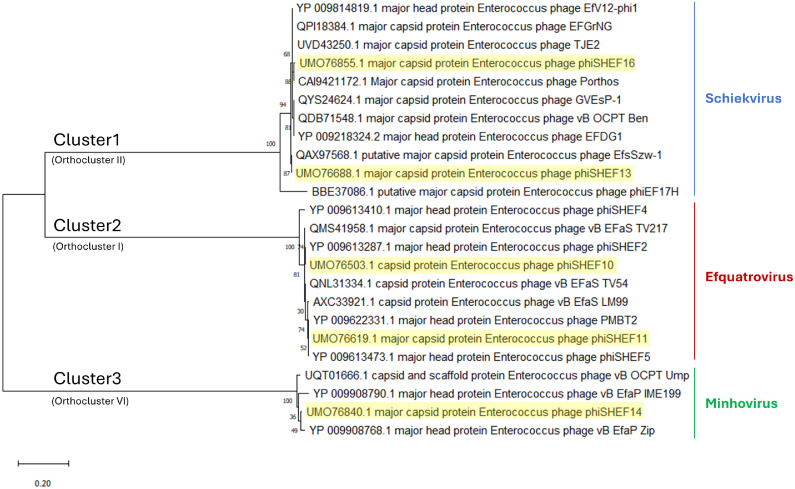
Phylogenetic trees comparing Major capsid protein sequences of the isolated phages with their closest corresponding in the database. The tree of Major capsid protein sequences was created using neighbour-joining method. Sequences were found to be classified based on phage morphology and ICTV genomic classification: (Cluster1 = Myoviruses, Schiekvirus, Cluster2 = Siphoviruses, Efquatrovirus and Cluster3 = Podoviruses, Minhovirus).

The genomes of the five isolated phages (SHEF10,11,13,14 and 16) were sequenced at MicrobesNG using the Illumina NovaSeq 6000 platform and assembled using SPAdes v3.7. The sequences were annotated using Prokka and RASTtk. As expected from their morphology, the phages had varied genome sizes summarised in Table 3. Bacteriophages SHEF10 and 11 are Efquatroviruses with genome sizes of 41.6 and 40.7 kb, respectively, while SHEF 13 and 16 belong to the Schiekvirus genus with genomes of approximately 150 kb. In contrast, SHEF14 is a member of the Minhovirus genus, with a genome of only 19.3 kb. Upon genome comparison using the NCBI non-redundant nucleotide database SHEF10, 11,13 and 16 were shown to be new variants of the same species as their closest matches, while SHEF14 looks to be a new species within genus Minhovirus, i.e. less than 95 % match to closest relative (Turner et al., 2021). To delve deeper into these relationships, phylogenetic trees were constructed using the major capsid proteins (MCP) (Fig. 2) and whole genome sequences (S5), revealing that these phage cluster with their genus relatives and morphological types into three clusters: cluster 1, Schiekvirus (myo); cluster 2, Efquatrovirus (sipho) and cluster 3, Minhovirus (podo) (Fig. 2). Furthermore, according to the Orthoclustering scheme suggested by Bolocan et al., these clusters are equivalent to Orthoclusters II, I and VI, respectively (Bolocan et al., 2019) (Fig. 2).

**Table 3:** Genomic characterisation of the isolated phages.

SHEF	ICTV classification	Size (kb)	CDS	GC%	Closest hit (identity%)
10	Efquatrovirus (genus), SHEF2 (species)	41.6	67	35 %	phiSHEF2 (99.61 %)
11	Efquatrovirus (genus), SHEF4 (species)	40.7	63	35 %	phiSHEF4 (98.84 %)
13	Herelleviridae (family) Schiekvirus (genus)	151.3	219	37 %	EfsSzw-1 (96.99 %)
14	Rountreeviridae (family); Sarlesvirinae (subfamily); Minhovirus (genus)	19.3	22	35 %	vB_OCPT_Ump (92.36 %)
16	Herelleviridae (family) Schiekvirus (genus)	152.9	185	37 %	Porthos (97.17 %)

**Fig. 3:**
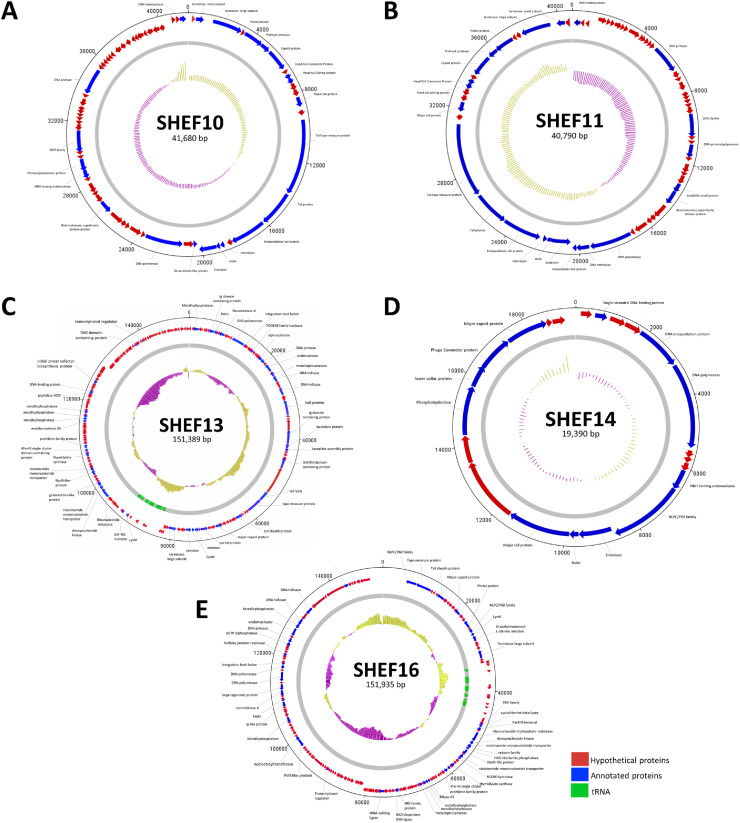
Circular Maps of SHEF genomes. The annotated genomes of (A) SHEF10, (B) SHEF11 and (C) SHEF13, (D) SHEF14 (E) SHEF16 are shown. Hypothetical proteins are labelled with red colour, annotated genes with blue with Blue and tRNA with green. The map was generated by using DNAPlotter from Artemis version 18.2.0.

Further investigation of the phage genomes revealed that all genomes were arranged in the typical modular fashion for their respective genera. All encoded a major capsid protein gene- predicted to possess the conserved HK97 fold (using Phyre2) (Duda and Teschke, 2019) (ORFs: SHEF10_7, SHEF11_56, SHEF13_63, SHEF14_20, SHEF16_13). In contrast, the tail modules differed among the isolated phages according to the observed virus morphologies. For example, the contractile SHEF13 and 16 possessed tail sheath proteins that are absent from the non-contractile SHEF10,11 and 14. As expected, a predicted tape measure protein (TMP) was also lacking in the non-tailed SHEF14 phage (Fig. 3). Analysis of predicted tail-associated lysins (TALs) revealed that SHEF10 and 11 possess proteins with predicted endopeptidase activities (SHEF10_17 and SHEF11_46). The myoviruses SHEF13 and 16 harbour two TALs: New Lipoprotein C/Protein of 60-kDa (NLPC/P60) (SHEF13_50, SHEF16_1) as well as lytic transglycosylases (SHEF13_51, SHEF16_2). For SHEF14, A predicted NLPC/P60 domain (SHEF14_11) was found within a protein situated between a HNH homing endonuclease and the endolysin proteins. According to our NLPC/P60 classification from enterococcal phages (Alrafaie and Stafford, 2022), SHEF14_11 belongs to Group1C while both SHEF13_50, SHEF16_1 group in Group2A (Fig. S1). Unsurprisingly, all our phages possessed DNA polymerase genes while only the more complex DNA metabolism modules of the Myoviruses (SHEF13 and16) containing predicted metallophosphatases and tRNAs. Notably, the genomes of SHEF phages lack genes encoding integrase and repressor genes, bioinformatically confirming our observation that they are strictly lytic viruses.

**Fig. 4:**
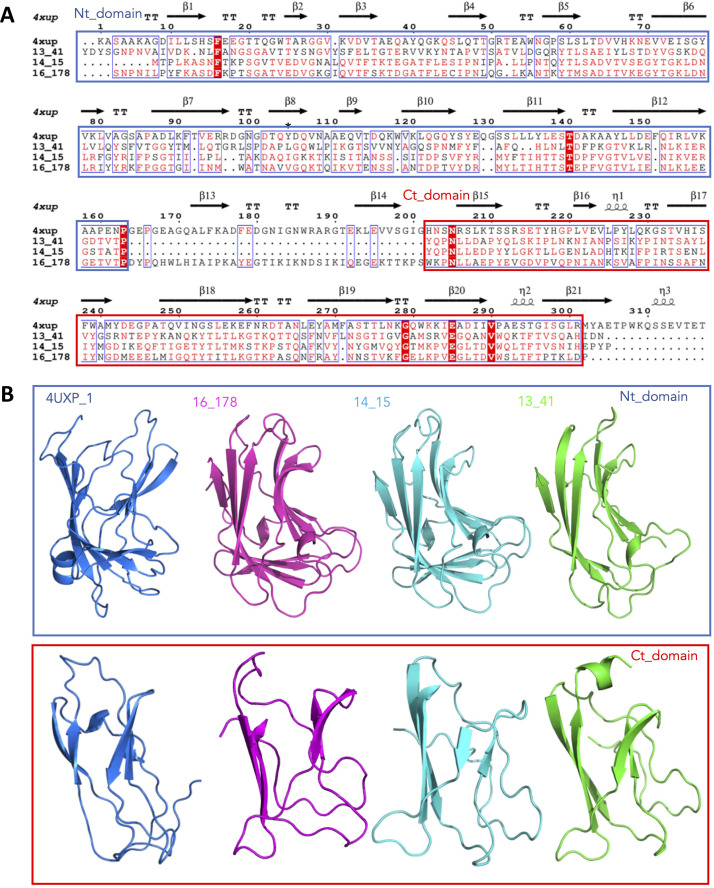
Conserved regions of Phage tail proteins containing a putative Carbohydrate binding module. A) Amino acid alignment of SHEF14_15, 13_41 and 16_178 with the CBM22 domain from *Paenibacillus barcinonensis* (4XUP) made using Multalin and 4XUP as the template for imposition of secondary structure motifs. B) Alpha fold models of the N and C terminal domains of SHEF14_15, 13_41 and 16_178 and the derived model of the CBM22 4XUP.

We then examined the genomes of the three phage that target VRE-E1071 (SHEF13,14,16) to look for commonalities that might explain the partially shared host-range. Sequence and structural predictions (PHYRE 2) of the tail-module proteins revealed the presence of a conserved domain of around 300 amino acids within the centre of putative tail proteins SHEF14_15, 13_178 and 16_41, (Fig. 4A, S3). Structural models built using alpha-fold showed that the central regions from all three proteins were comprised of two anti-parallel beta stranded sub-domains separated by a linker region (Fig. 4B and S4). The models of the N-terminal and c-terminal portions structurally align both with each other and with the CBM domains of a xylanase from *Paenibacillus barcinonensis* (PDB model 4XUP) that is predicted to encode a sugar-binding domain of the CBM22 tandem domain family (CAZy**,** (Drula et al., 2022)). These and related CBM domains have been shown in various organisms to bind carbohydrate polymers (Devillard et al., 2003; Obembe, 2009; Sainz-Polo et al., 2015). In the context of phage and enterococci, this may suggest targeting of carbohydrate structures on bacterial surfaces such as those within EPA (Guerardel et al., 2020).

**Fig. 5:**
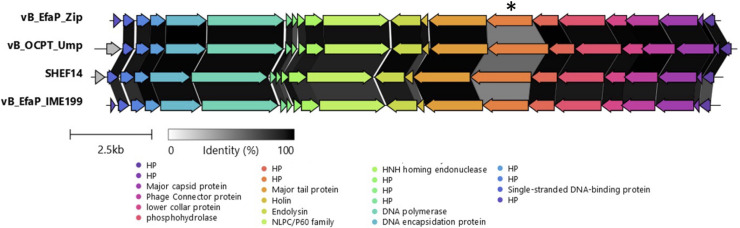
Multiple Sequence Alignment of vB_EfaP_Zip, vB_OCPT_Ump, SHEF14 and vB_EfaP_IME199. The Asterisk indicates the location of SHEF14_15 and its corresponding CDSs Scale bar and identity percentage are also shown.

As outlined above, SHEF14 is a member of small genome minhovirus that have differing host-ranges. Comparison of the genome sequences of four of the most closely related of these (vB_EfaP_Zip, vB_OCPT_Ump, SHEF14 and vB_EfaP_IME199) (Fig. 5) shows highly conserved genomes with a region of divergence in the SHEF14_15 ORF sequence and homologues. In contrast to SHEF14 and UMP which both contain the CBM 22 domain, the ORF15 homologues within vbEFA_ZIP and IME199 lack this inserted domain. We postulate that this domain may define host-specificity in these elegantly minimal *Enterococcus* minhoviruses and also possibly in the other viruses infecting E1071, given its shared presence in the Schiekviruses isolated in this study.

### 3.3 Role of the Enterococcus EPA_var region in virus-host interactions

**Fig. 6:**
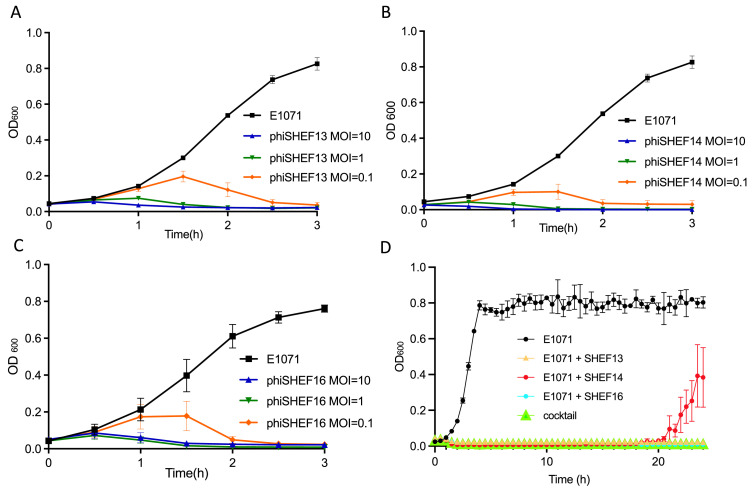
Killing assays of SHEF13, 14 and 16 (A-C). The different MOIs are indicated by colours: blue (MOI 10), green (MOI 1) and orange (MOI 0.1). D: 24 h Incubations of E1071 with phage as indicated, at MOI 0.1. Experiments were performed in biological triplicate (with 3 wells per condition), Error bars represent SEM.

We next focused in detail on the three phages targeting *E. faecium* strain E1071. Killing assays with SHEF13,14,16 and a cocktail of all phage with strain E1071 showed rapid killing at different MOIs (10, 1 and 0.1) after as little as 30 mins (Fig. 6A-C). We then performed the experiments over 24 h at two different MOI (1 and 0.1) to ask whether either the single phage alone, or the cocktail of all three phage would allow the emergence of resistance. As shown in Fig 6D(and S6) the OD600 of the cultures was suppressed for 24 h by SHEF13 and SHEF16, while SHEF14 cultures rebounded at around 14 (MOI=1) and 21 h (MOI=0.1). Hence we conclude that a cocktail of our phage can suppress growth of the VRE strain E1071, with a lower MOI being more effective.

**Fig. 7:**
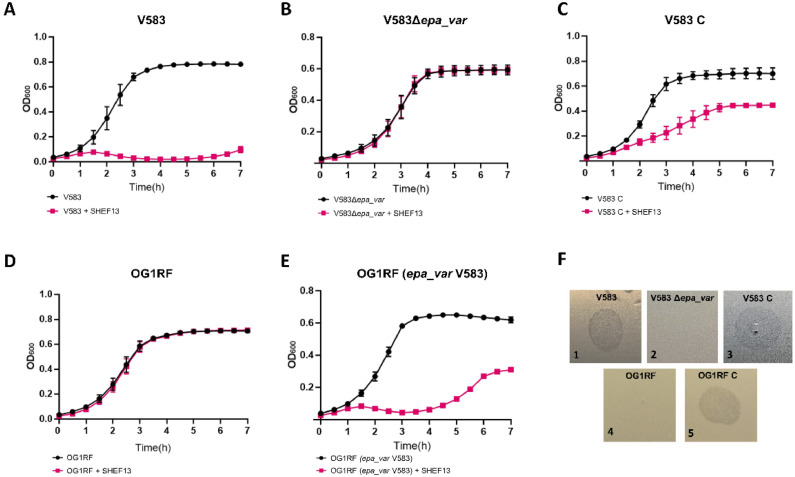
The effect of the V583 EPA decorations on SHEF13 infection. Killing assays were carried out with SHEF13 (MOI=1) and OD_600_ was measured in the absence (black lines) or presence (red lines) of the phage. The strains used were V583 (A), V583 D*epa_var* (B); V583 D*epa_var* (epa_varV), a V583 D*epa_var* mutant complemented with a plasmid-encoded *epa_var* region from V583 (C); OG1RF (D); OG1RF D*epa_var* mutant complemented with a plasmid-encoded *epa_var* region from V583 (E). This experiment was done in triplicate and error bars represent SEM for three replicates. (F) Spots tests of SHEF13 on bacterial lawns in which lysis indicates a positive result.

In enterococci, EPA is frequently targeted during phage adsorption (Al-Zubidi et al., 2019; Ho et al., 2018; Teng et al., 2009). EPA is made of two polysaccharidic chains: a rhamnose backbone encoded by a set of 18 conserved genes (*epaA-R*) and phosphate-containinig decorations encoded by a variable region (*epa_var*) that are strain-specific (Guerardel et al., 2020). We investigated the role played by EPA decorations in the infection by SHEF13 using two *E. faecalis* strains encoding distinct decorations loci (V583 and OG1RF) and the V583 derivatives previously described (Furlan et al., 2019). As opposed to V583 (Fig. 7A, F1), the isogenic mutant with a complete 17 kbp deletion of the *epa_var* locus could no longer be infected by SHEFF13 (Fig. 7B, F1, zero plaques with 2 × 10^7^ PFU/ml). Infection could be restored by complementation with the expression of the *epa_var* locus on the low copy replicative plasmid pIL252 (Fig. 7C, F3). Whilst SHEFF 13 was unable to infect OG1RF (Table 2 and Fig. 7D, F4, zero plaques with 2 × 10^7^ PFU/ml input), the expression of the *epa_var* region of V583 in OG1RF harbouring a complete deletion of the *epa_var* region partially confers susceptibility to SHEFF13 as observed in growth experiments, but at an EOP of 100 compared to V583_WT in plaque assays (2 × 10^9^ pfu/ml from 2 × 10^7^ PFU/ml input). Collectively, these data indicate that the recognition of V583 EPA decorations by SHEFF13 is necessary for the lytic cycle of this phage.

We next examined the loci encoding the EPA decorations in a range of *Enterococcus faecium* and *faecalis* strains. *E. faecalis* V583 consists of 13 genes running from *ef2176* to *ef2164* (Guerardel et al., 2020). The OG1RF *epa_var* contains several genes with no homology with V583. These include *ef2175* (licD-related protein), *ef2172* (2-C-methyl-d-erythritol 4-phosphate cytidylyltransferase), *ef2171* (epimerase), *ef2168* (licD1 protein), *ef2167* (glycosyl transferase) and *ef2166* (membrane protein). One or more of these genes are potentially involved in EPA modifications influencing bacterial susceptibility to SHEF13. To further investigate this, the EPA variable regions were further compared with those of SHEF13 susceptible (DP7, DP9, V583, E1071) and resistant (OG1RF, DP5, E4452) strains (Fig 6). When examining the presence or absence of genes in relation to susceptibility to SHEF13, the OG1RF/ DP5 genes OG1RF_1171111709 and DP5_1846,1845 appear to have no homologues in *E. faecalis* V583 or any of the *E. faecium* strains infected by SHEF13 (E1071,DP7,DP9). OG1RF_1171111709 putatively encode EPA polymerization proteins and possibly suggest a modification that might block phage interaction. Notably, some CDSs were only found among *E. faecalis* genomes, such as the predicted glycosyl transferase OG1RF_11720 (DP5_1853, V583_2176) suggesting species specific modifications, although at this stage we do not know how these influence bacterial surface recognition.

## 4 Discussion

This study focused on isolating phages targeting VRE *E. faecium* and *faecalis* strains with the potential to act as therapeutic agents against antibiotic-resistant enterococci, particularly in individuals with conditions like diabetic foot ulcers (DFU). Five novel phages (SHEF10,11,13,14 and 16) were isolated. However, isolation against the VRE strain E1071 was only successful using the multiple-host method (Oliveira et al., 2009; Sillankorva et al., 2010). We also were unable to isolate phages from the other two EPA loci variants (V3-V4) in this study and this is an ongoing focus.

**Fig. 8:**
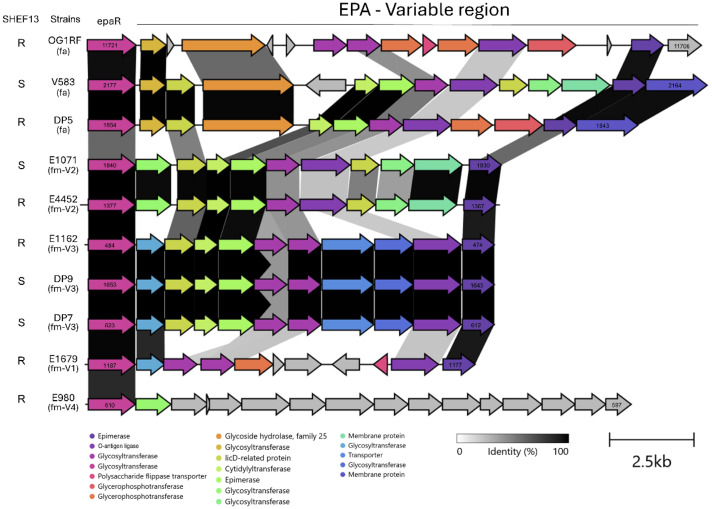
Alignment of enterococcal EPA variable regions from *E. faecalis* OG1RF, V583 DP5 and *E. faecium* E1071, E4452, E1162, DP9, DP7, E1679 and E980 were aligned. fa refers to faecalis while fm to faecium. V1–4 indicate epa_var type defined by De Been et al., Scale bar and identity percentage are also shown.

Our study isolated several siphovirus like phage infecting *E. faecalis* that had some homology to phage previously isolated by our group (Al-Zubidi et al., 2019). In contrast when using *E. faecium* as a host for isolation, we isolated a group of diverse myovirus (schiekvirus) and podovirus (minhovirus), namely SHEF13,14 and 16. One of these, SHEF13 displayed a broad host-range infecting both *E. faecium* (EPA- V2) and *E. faecalis* strains and a dependency on EPA decorations. The isolation of broad-host range myo-like phages with a broad host-range has been previously reported with the closely related EFDG1 (92 %) (Khalifa et al., 2015), as well as vB_OCPT_Ben (Wandro et al., 2022) and others (D'Andrea et al., 2020; Del Rio et al., 2019). In contrast SHEF16 infects strains belonging to both variants 1 (E1679 and E1636) and 2 (E1071 and E4452), suggesting the existence of a common receptor in these strains for SHEF16 infection. However, the picture may well be more complex as SHEF13, which our data show needs EPA for infection, was unable to infect the other EPA_V2 strain in our collection, E4452, despite a closely related *epa_var* locus (Fig 8). Of note there are clearly shared genes between *E. faecium* EPA_V2 and V583, but not OG1RF. Elucidating the structure of EPA in these strains is therefore required to understand the molecular basis of surface recognition by SHEF13.

However, it is likely that EPA is not the sole determinant of adsorption, as illustrated by both the gene loci comparisons but also work by other groups indicating that phage resistant strains of *Enteroccus* spp. accumulate mutations in the EPA genes, but also in DNA replication and RNA translation genes (Canfield et al., 2021; Chatterjee et al., 2019, 2020; Wandro et al., 2022). With our phage set, we do observe phage resistance after prolonged incubation (Fig 6D), but have not yet fully characterized these strains or their impact on virulence or AMR profiles, but we have preliminary data that despite the lack of infection in the absence of the EPA_var genes the phage still adsorb to the cell surface (not shown), but the mechanism of how this occurs will be a future focus of our work.

All of the phages described in this study displayed a lytic life-cycle, i.e. lacked lysogenic genes, but also contained considerable numbers of genes with hypothetical function, highlighting the need for further studies to clarify their functions in phage-host interactions. A case in point is SHEF14 which has a genome of 19.3 kb, with only 22 predicted coding genes but still with 4 genes with no predicted function. It is worth noting that SHEF14 and other podoviruses that have ability to infect and kill AMR pathogens despite their small genetic complement make them excellent candidates for evolution or engineering (Mahler et al., 2023) and advocates for their use in phage cocktail design. In this context, our potential VRE cocktail of SHEF13,14 and 16; which rapidly suppress *E. faecium* populations may allow development of an effective cocktail, especially if mixed with a wider range of diverse phage.

Finally, as well as considering the characteristics of the host EPA locus, our bioinformatic analysis of three phage infecting the same VRE strain (E1071) uncovered the presence of a shared domain in the SHEF14_15; SHEF16_178 and SHEF13_41 protein sequence, which is also shared in other viruses like vB_OCPT_Ump. The predicted function of this domain, based on sequence and structural modelling (using alpha-fold), is that it is a Carbohydrate binding module (CBM). While it is tempting to surmise that this binds sugar polymers in a manner similar to the closest homologue of a xylanase containing the CBM22 domains binding xylans (Sainz-Polo et al., 2015), the absence of conserved ligand binding residues in the 14_15 group means a different substrate may be the target, possibly a rhamnose polymer that is part of the EPA (Guerardel et al., 2020). Our group is currently investigating the function of SHEF14_15.

In conclusion, the identification and characterization of SHEF phages provide important novel knowledge about their host specificity and genomic characteristics. These findings provide the basis for additional investigation into the therapeutic potential of these phages. Furthermore, the finding of specific genes linked to phage susceptibility, such as those in the EPA variable area, opens up interesting opportunities for developing focused strategies for phage therapy as well as understanding the fundamental biology of host-phage interactions in enterococci. This work has great potential to make a major contribution to the development of treatments for enterococcal infections, particularly antibiotic-resistant strains.

## CRediT authorship contribution statement

**Alhassan M. Alrafaie:** Writing – review & editing, Writing – original draft, Methodology, Investigation, Formal analysis, Data curation, Conceptualization. **Karolina Pyrzanowska:** Writing – review & editing, Investigation. **Elspeth M. Smith:** Investigation, Writing – review & editing. **David G. Partridge:** Writing – review & editing, Resources, Investigation. **John Rafferty:** Writing – review & editing, Formal analysis. **Stephane Mesnage:** Writing – review & editing, Visualization, Resources, Methodology, Investigation, Conceptualization. **Joanna Shepherd:** Writing – review & editing, Supervision, Funding acquisition. **Graham P. Stafford:** Writing – review & editing, Writing – original draft, Visualization, Supervision, Resources, Project administration, Methodology, Investigation, Formal analysis, Data curation, Conceptualization.

## Declaration of competing interest

The authors declare that they have no known competing financial interests or personal relationships that could have appeared to influence the work reported in this paper.

## Data Availability

Data will be made available on request.

## Appendix Supplementary materials

Image, application 1
