# Supplementary material for: A diverse set of Enterococcus-infecting phage provides insight into phage host-range determinants
Source: Virus Res. 2024 Jul 4;347:199426. doi: 10.1016/j.virusres.2024.199426 (PMC11269942; doi:10.1016/j.virusres.2024.199426)
Supplement: Supplementary file 1 [file mmc1.pdf]

Supplementary data

Table S1. list of enterococcal strains used in this study.

Strain	Species	Phage infection
DP1	*E. faecalis*	SHEF13
DP2	*E. faecalis*	-
DP3	*E. faecalis*	SHEF13
DP4	*E. faecalis*	-
DP5	*E. faecalis*	SHEF10
DP6	*E. faecium*	-
DP7 (EPA_V3)	*E. faecium*	SHEF13
DP8	*E. faecium*	-
DP9 (EPA_V3)	*E. faecium*	SHEF13, 16
E1636 (EPA_V1)	*E. faecium*	SHEF16
E1679 (EPA_V1)	*E. faecium*	SHEF16
E1071 (EPA_V2)	*E. faecium*	SHEF13,14,16
E4452 (EPA_V2)	*E. faecium*	SHEF16
E1162 (EPA_V3)	*E. faecium*	-
E980 (EPA_V4)	*E. faecium*	-
Aus0004 (EPA_V4)	*E. faecium*	-
U0317	*E. faecium*	-
EF1	*E. faecalis*	-
EF2	*E. faecalis*	SHEF11,13
EF3	*E. faecalis*	-
OS16	*E. faecalis*	SHEF10
OMGS3919	*E. faecalis*	SHEF10
EF54	*E. faecalis*	SHEF10
JH2-2	*E. faecalis*	SHEF13
OG1RF	*E. faecalis*	SHEF10
OG1RF ΔepaV + pIL-epaV (V583)	*E. faecalis*	SHEF13
V583	*E. faecalis*	SHEF13
V583 ΔepaV	*E. faecalis*	-
V583 ΔepaV + pILepa-V	*E. faecalis*	SHEF13
R178	*E. faecalis*	-
R197	*E. faecalis*	-
R51	*E. faecalis*	-
R5	*E. faecalis*	-
K2756-02	*E. faecalis*	-
R53	*E. faecalis*	-
R70	*E. faecalis*	-
ATCC 51575	*E. faecalis*	-
ATCC 51299	*E. faecalis*	SHEF13
ATCC 19433	*E. faecalis*	SHEF11,13
ATCC 29212	*E. faecalis*	SHEF10
8413	*E. faecalis*	SHEF10,13

Table S2. Genomic characterisation of the DP enterococcal clinical isolates.

Species	Strains	Size (Mb)	Genotype	Predicted phenotype	MLST
E. faecalis	DP1	3.07	aac(6')-aph(2"), ant(6)-Ia, aph(3')-III, erm(B), lsa(A), tet(M)	amikacin, gentamicin, tobramycin, streptomycin, kanamycin, erythromycin, azithromycin, lincomycin, tetracycline	179
	DP3	3.04	aac(6')-aph(2"), ant(6)-Ia, aph(3')-III, erm(B), lsa(A), tet(M)	amikacin, gentamicin, tobramycin, streptomycin, kanamycin, erythromycin, azithromycin, lincomycin, tetracycline	179
	DP5	2.94	cat, dfrG, erm(B), lsa(A), str, tet(M)	chloramphenicol, trimethoprim, erythromycin, azithromycin, lincomycin, streptomycin, tetracycline	16
E. faecium	DP7	3.08	aac(6')-Ii, ant(6)-Ia, aph(3')-III, erm(B), msr(C), tet(M	amikacin, tobramycin, streptomycin, kanamycin, erythromycin, azithromycin, tetracycline	17
	DP9	3.11	aac(6')-aph(2"), aac(6')-Ii, ant(6)-Ia, ant(9)-Ia, aph(3')-III, dfrG, erm(A), erm(B), lnu(B), lsa(E), msr(C), tet(M), VanHAX	amikacin, gentamicin, tobramycin, streptomycin, spectinomycin, kanamycin, trimethoprim, erythromycin, azithromycin, lincomycin, unknown[lsa(E)_1_JX560992], tetracycline, vancomycin	787

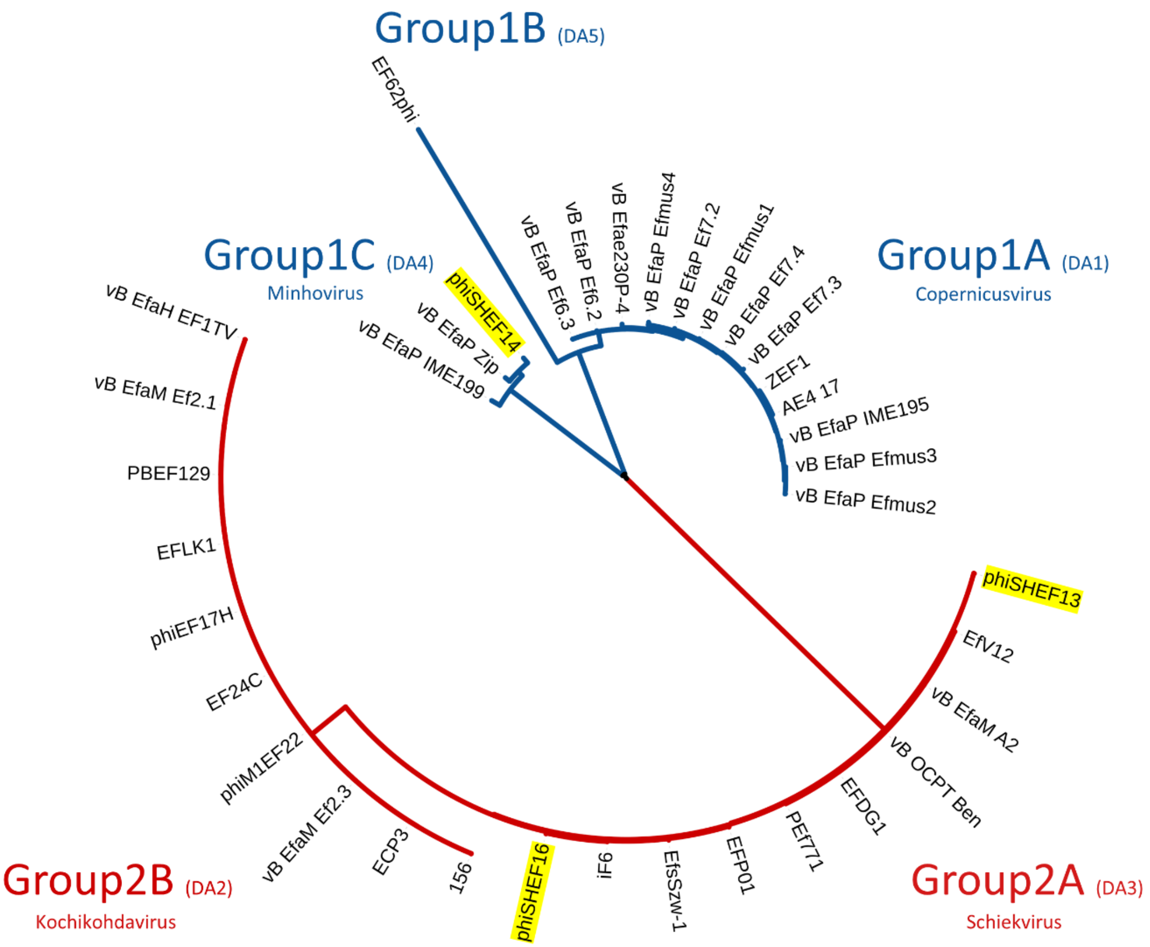

Fig S1. A phylogenetic tree of enterococcal NLPC/P60 containing proteins. This shows two main groups based on phage genomic classification and morphology: sequences from podoviruses labelled Blue and myoviruses

labelled red. NLPC/P60 sequences from SHEF13, 14 and 16 were labelled with yellow. The tree was constructed using FastTree and visualised using the ITOL online website. Adapted with modification from (Alrafaie & Stafford, 2022)

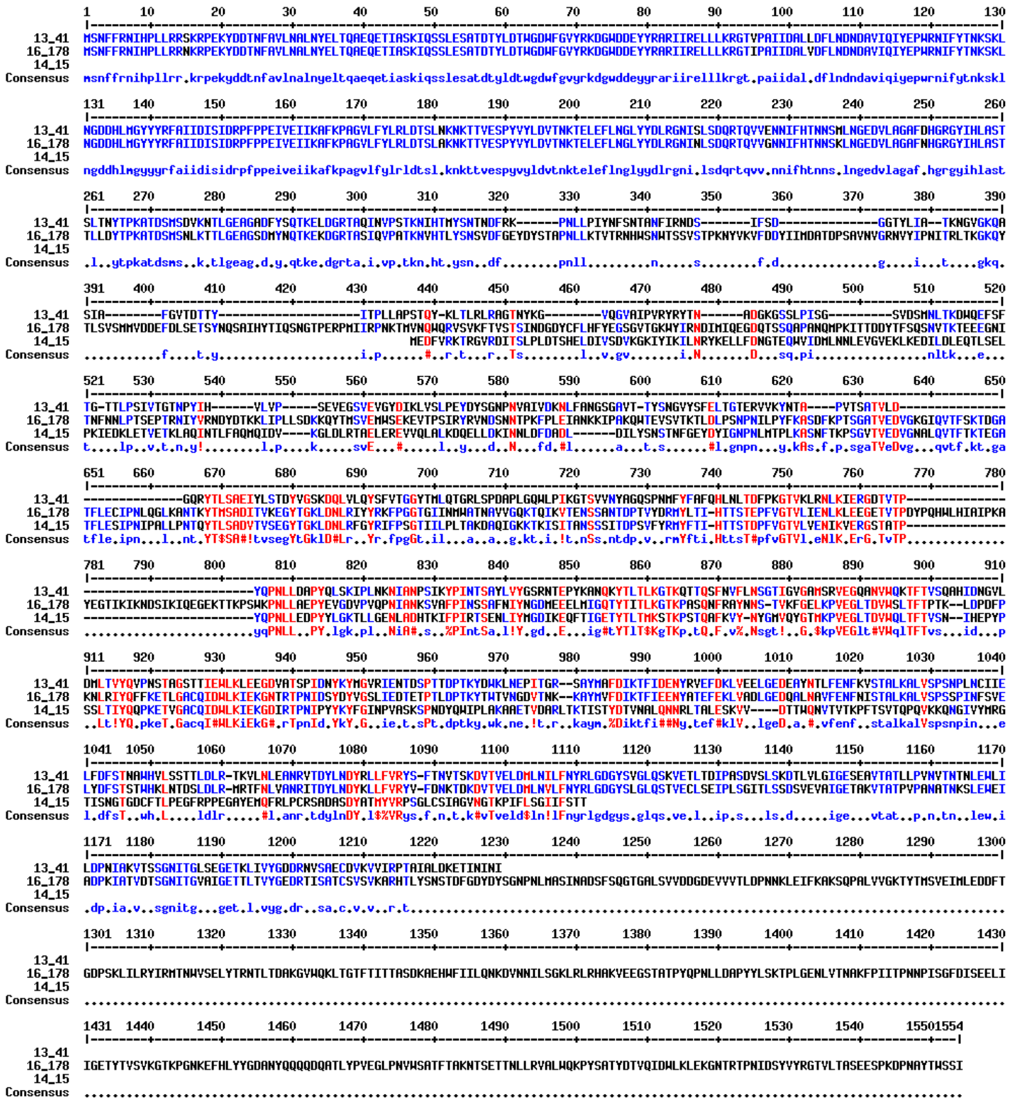

Fig S2. Multiple sequence alignment of three coding sequences: SHEF13_41, SHEF14_15 and SHEF16_178. The alignment showed high consensus (red font) between the three proteins at the central region (440-1110aa).

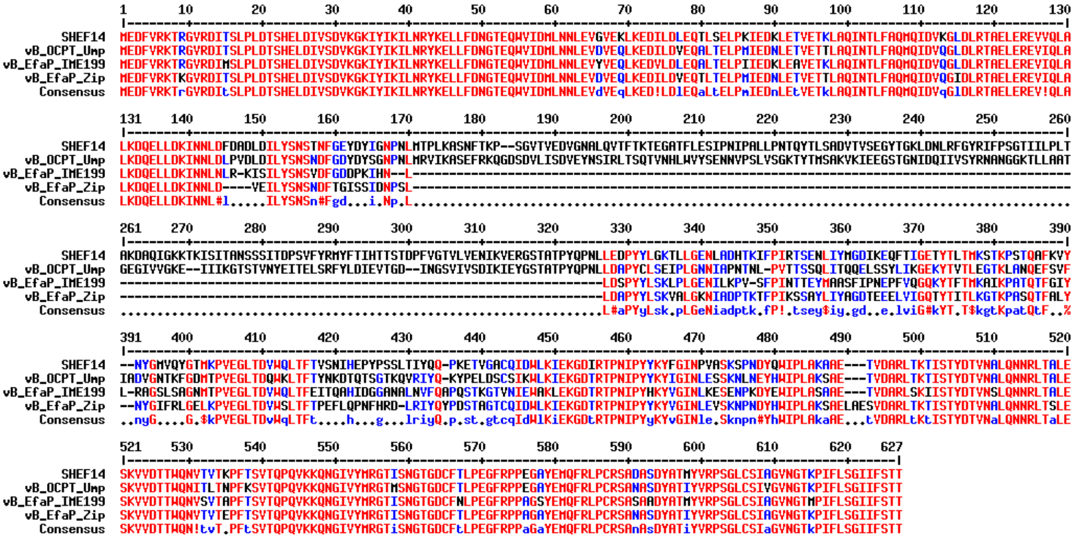

Fig S3. Multiple sequence alignment of coding sequences: SHEF14_15, vB_OCPT_Ump_UQT01671.1, vB_EfaP_IME199_ALO81016.1 and vB_EfaP_Zip_gp08. The alignment showed high consensus (red font), low consensus (blue) and neutral (black) between the analysed proteins.

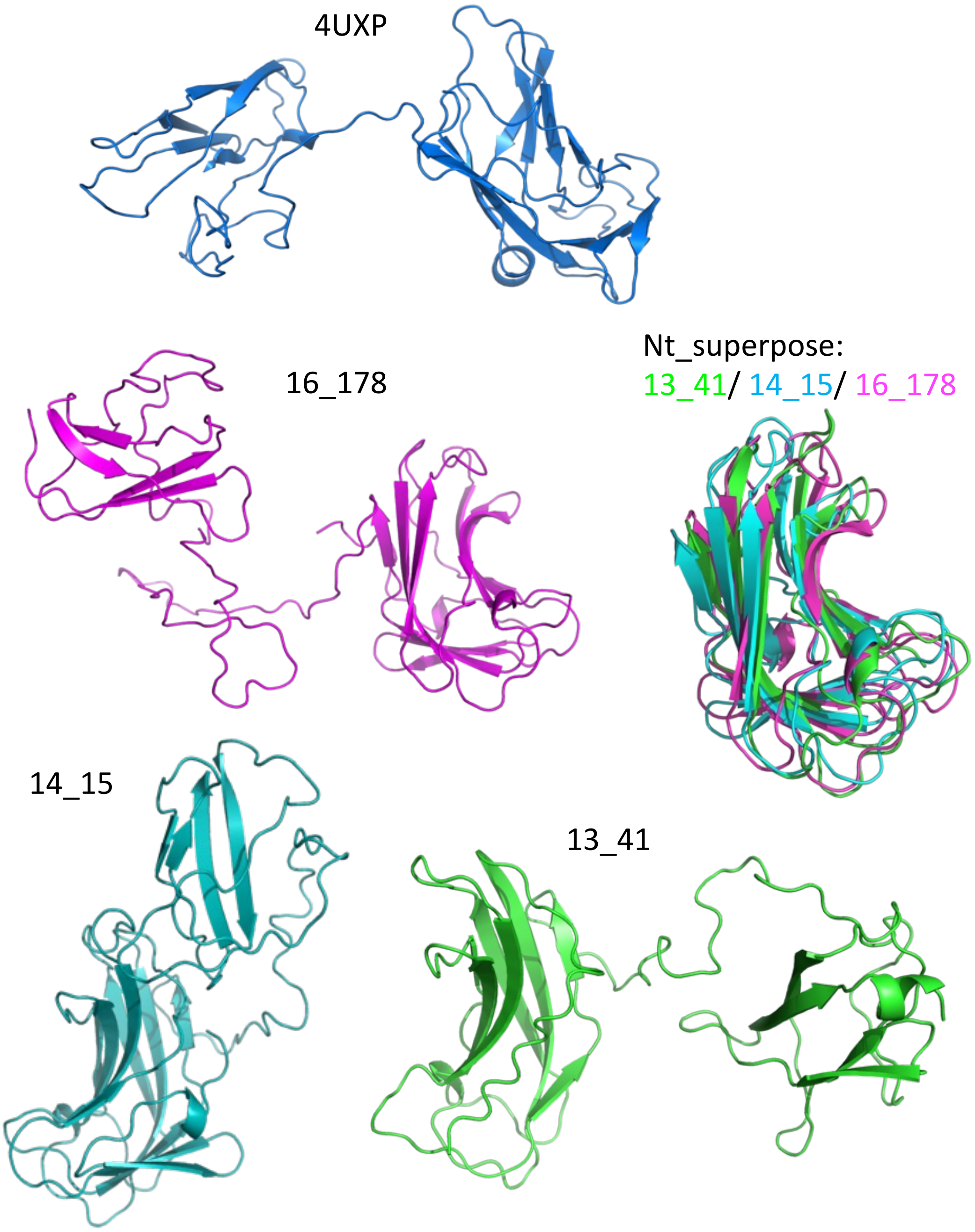

Fig S4. Structural models of phage proteins from alpha-fold, with orientation according to the N-terminal domain and a superposition of all four N-terminal domains.

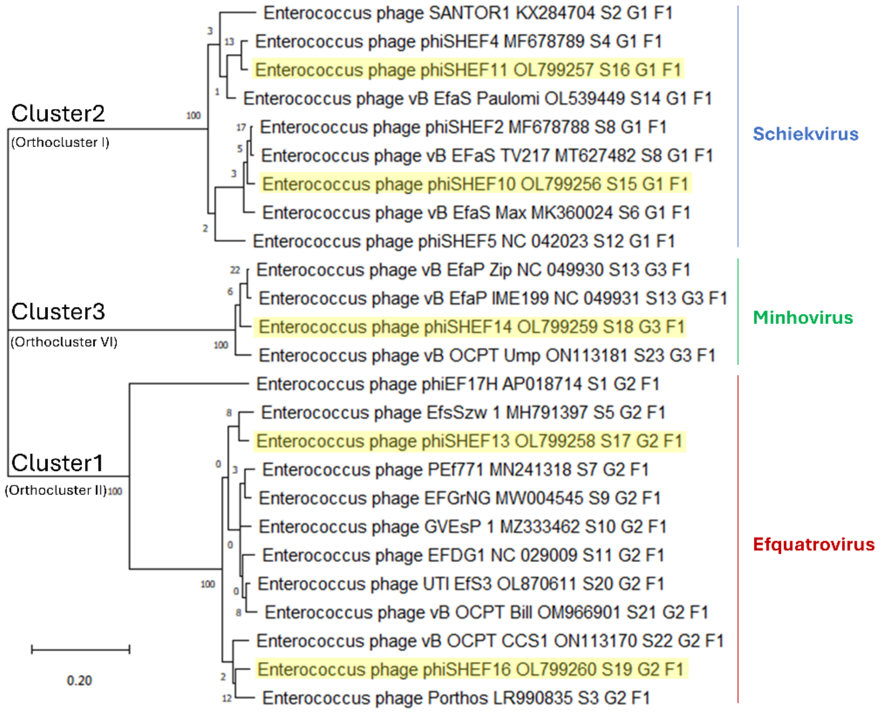

Fig S5. Phylogenetic trees comparing whole genome sequences of the isolated phages with their closest corresponding in the database. The tree of whole genome sequences was created using the Genome-BLAST Distance Phylogeny (GBDP) method via VICTOR. Sequences were found to be classified based on phage morphology and ICTV genomic classification: (Cluster1= Myoviruses, Schiekvirus x, Cluster2= Podoviruses, Minhovirus and Cluster3= Siphoviruses, Efquatrovirus).

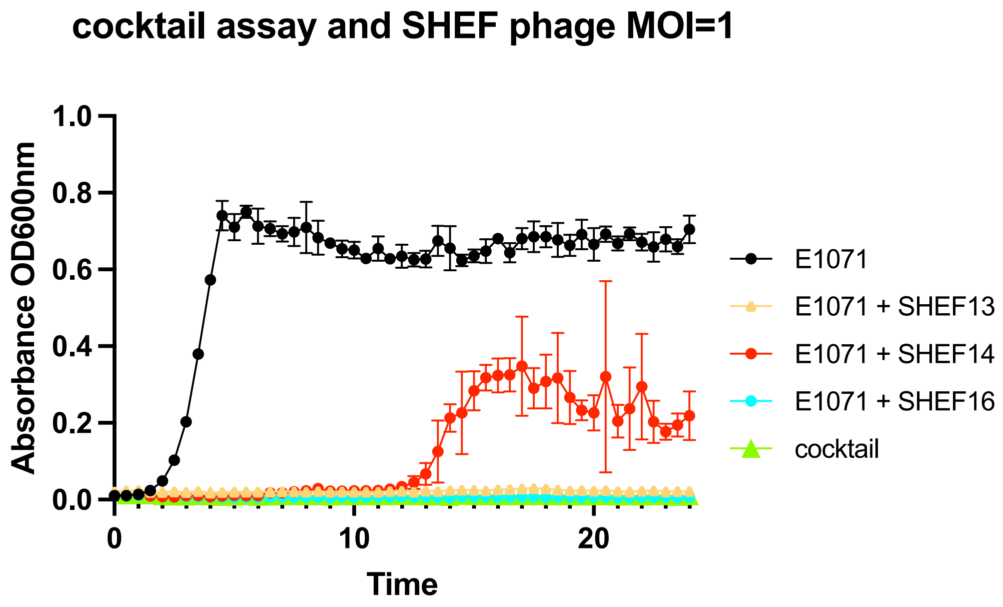

Fig.S6. 24h Incubations of E1071 with phage as indicated, at MOI 0.1.
Experiments were performed in biological triplicate (with 3 wells per condition), Error bars represent SEM.
